# Supplementary material for: Analytical Characterization of the Intercalation of Neutral Molecules into Saponite
Source: Molecules. 2022 May 10;27(10):3048. doi: 10.3390/molecules27103048 (PMC9143227; doi:10.3390/molecules27103048)
Supplement: Supplementary file 1 [file molecules-27-03048-s001.zip › molecules-1696944-supplementary.pdf]

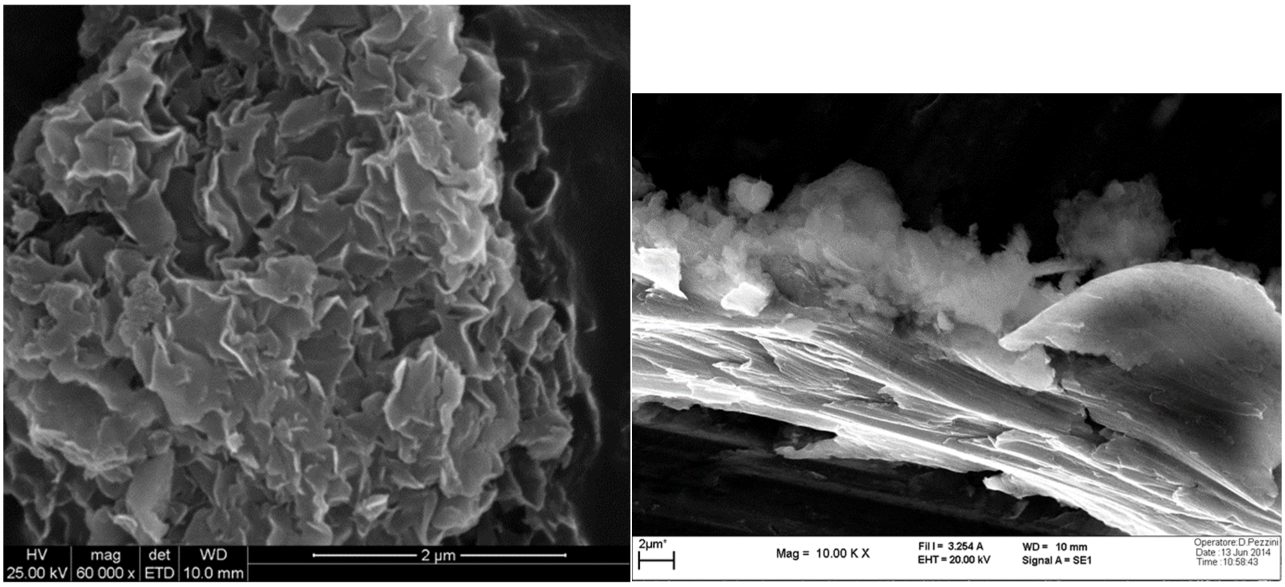

Figure S1. SEM image of CTA_Sap_LAG (left) and CTA_Sap_OP (right)

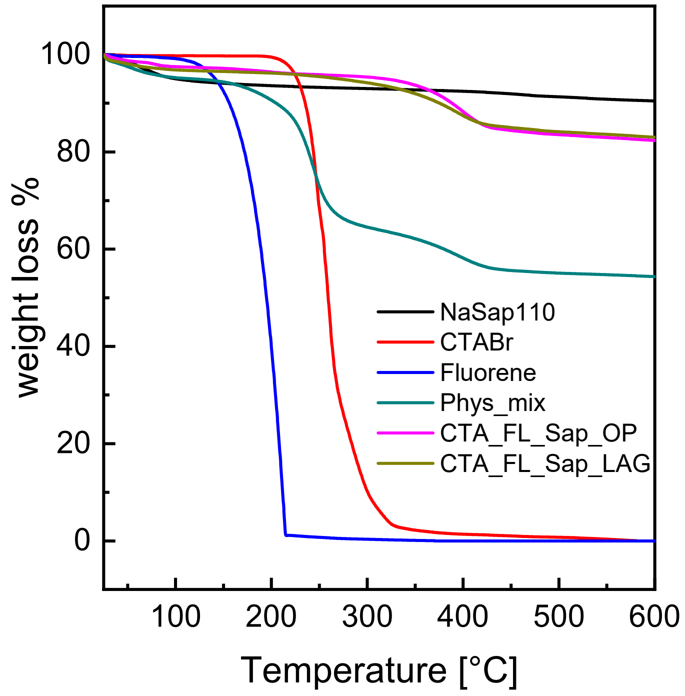

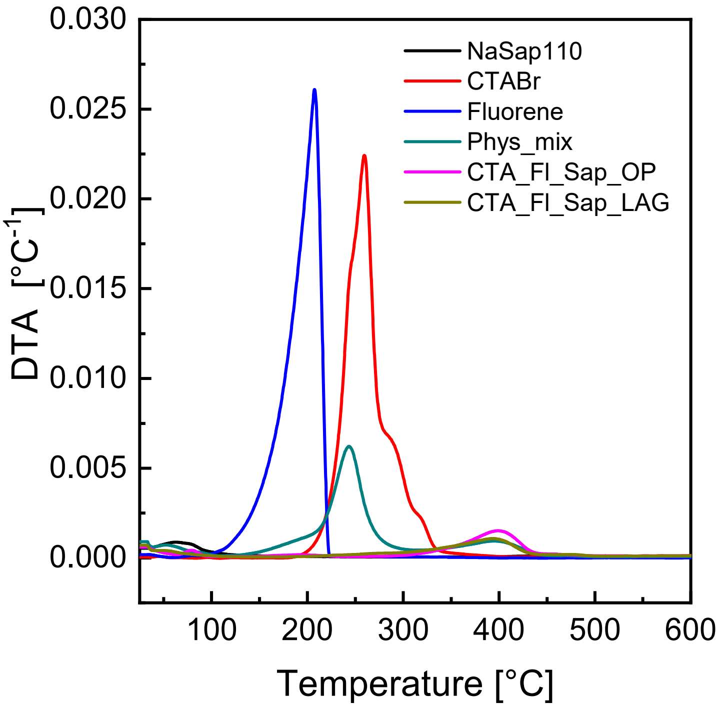

Figure S2. TGA and dTA curves at 10°C/min under helium flow for Na-Sap110 (black line), CTAB (red line), fluorene (blue line), physical mixture (dark cyan line), one-pot intercalated saponite (magenta line) and LAG intercalated saponite (dark yellow line).

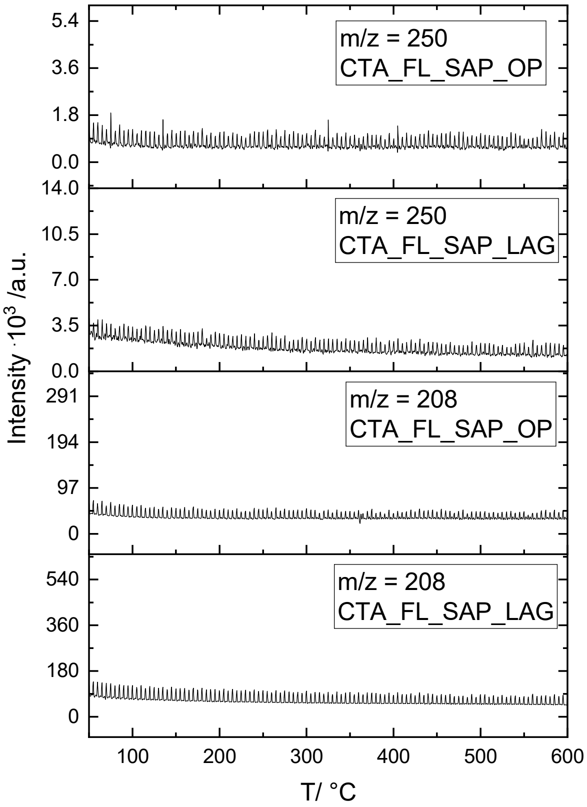

Figure S3. Evolution profiles of the signal at 250 m/z and 208 m/z recorded in the analyses of the OP and LAG materials.

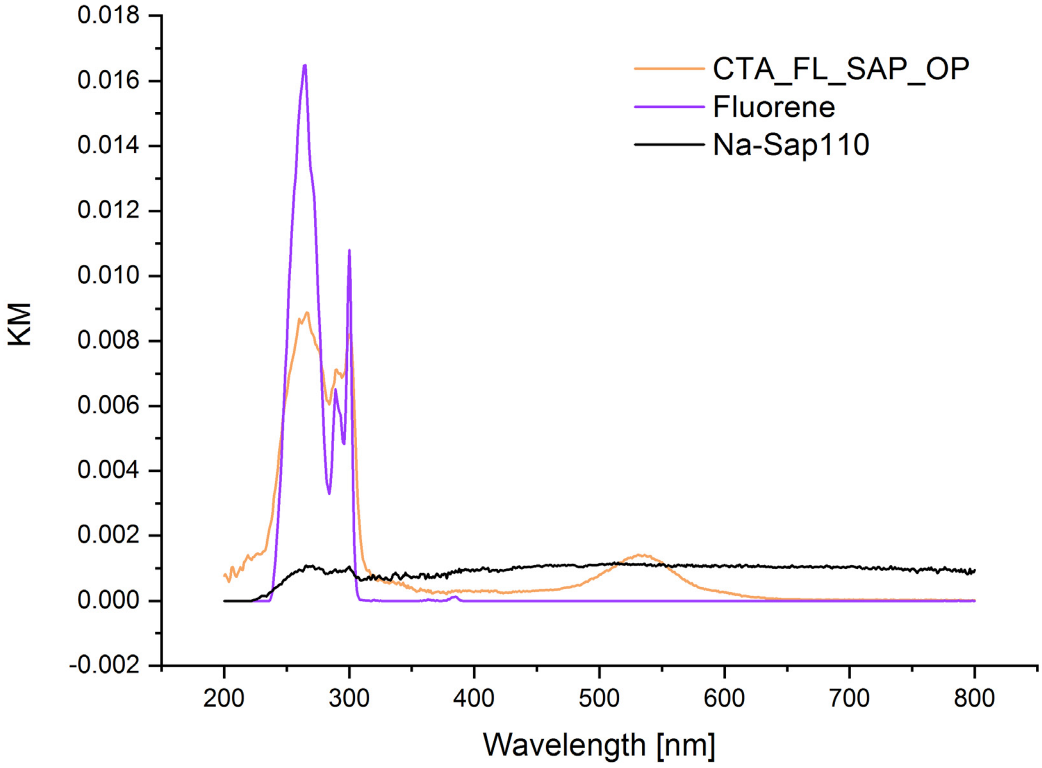

Figure S4. DR-UV/Vis spectra of CTA_FL_SAP_OP (yellow), Fluorene (violet) and Na-Sap110 (black).
